# A Forecast of Ophthalmology Practice Trends in Saudi Arabia: A Survey of Junior Residents

**DOI:** 10.4103/0974-9233.71606

**Published:** 2010

**Authors:** Fahad Alwadani, Aziz Alrushood, Hisham Altokhy, Tariq Alasbali

**Affiliations:** 1 Department of Ophthalmology, King Faisal University, King Fahad Hospital of the University, P. O. Box 2208, Al-Khobar, Saudi Arabia, 31952; 2 Department of Ophthalmology, Faculty of Medicine, McGill University, Montreal, Quebec, Canada

**Keywords:** Career Choice, Ophthalmology, Residency, Saudi Arabia, Specialties

## Abstract

**Purpose:**

The aim of this study is to identify the trends in practice pattern among current ophthalmology residents in Saudi Arabia.

**Materials and Methods:**

Ophthalmology residents in Saudi Arabia responded anonymously to a written survey between November 2007 and February 2008. The survey contained questions on demographic information, medical education, residency training, career goals and factors influencing their career choice. The data were categorized by gender. The influence of gender on outcome was assessed in a univariate fashion using the Chi-square or Fisher exact test when appropriate. A P-value of 0.05 or less was considered statistically significant for all analyses.

**Results:**

A total of 68 out of 85 residents (80%) responded to the survey. Over one-half of the residents preferred to pursue a fellowship within Saudi Arabia (53%), while others (25%) planned to train in North America. The majority of respondents wished to practice in an urban setting (63%). Anterior segment was the most desired subspecialty, while general ophthalmology and glaucoma were not a popular choice. Most residents were interested in refractive surgery (77%) and research (75%). The main factor influencing the decision to pursue ophthalmology was the ability to combine medicine and surgery (97%), while a positive elective experience was also an important factor, particularly for female respondents (91% vs. 57%; *P* < 0.001).

**Conclusion:**

Concerted efforts are required to encourage adoption to ophthalmic practice in public institutions rather than in private practice. In addition training in underrepresented subspecilaties should be encouraged to ensure adequate ophthalmic care for all citizens of Saudi Arabia.

## INTRODUCTION

The major provider of health care services in Saudi Arabia is the Ministry of Health (MOH). The MOH spends considerable effort providing modern ophthalmic care to its citizens, allocating as much as 10% of total health care resources to eye care.^1^ With the establishment of the King Khaled Eye Specialist Hospital (KKESH) in 1982, the modern ophthalmic care system in Saudi Arabia has made marked progress,^2^,^3^ decreasing the prevalence of blindness in the elderly by over 10% in a decade.^4^

However, there are regions in the country that underserved and other regions with relative surpluses of general ophthalmologists and ophthalmic subspecialists.^1^ Identifying the future plans of residents in training and motivational factors may provide an important insight into future delivery of ophthalmology and the changes required to meet the demands of ophthalmic care in Saudi Arabia.

The aim of this study is to identify trends and factors influencing the future practice plans of current ophthalmology residents in Saudi Arabia.

## MATERIALS AND METHODS

Anonymous questionnaires were sent to all ophthalmology residents in Saudi Arabia in November 2007. Residents were asked to complete the questionnaires, place it in a sealed envelope and return it to the study coordinator of their institution. The coordinators were given self-addressed stamped envelopes to mail the completed surveys by January 2008. If a response was not received by the deadline, coordinators were contacted via telephone and a reminder was sent to complete the questionnaire by February 2008. No compensation was provided to residents for completing the survey.

The survey contained questions regarding demographic information, medical education and residency training, career goals and factors influencing their career choice. A copy of the survey used in this study is presented in Table 1.

**Table 1:** Survey sample

Thank you for participating in this questionnaire		
Sex:	Age:	
Year of training:		
Province of training:		
Why you chose ophthalmology:
Yes No Major reason (**Choose one**)	
To combine medical and surgical:		
Intellectual stimulation/challenge:		
Workload flexibility and/or predictability:		
Positive elective experience:		
Positive undergraduate experience:		
Doctor-patient relationship:		
Earning potential (high income)		
Teaching opportunity		
Research opportunity		
Prestige		
Family influence:		
Others		
What is your future plan after training? Choose One
- Practice as a general ophthalmologist:	[ ]
- Fellowship in Saudi Arabia:	[ ]
- Fellowship in North America:	[ ]
- Fellowship in Australia:	[ ]
- Fellowship elsewhere:	[ ]
- Undecided:	[ ]
If you consider fellowship, which area of training: Choose one
- Anterior segment:	[ ]
- Medical retina:	[ ]
- Surgical retina:	[ ]
- Glaucoma:	[ ]
- Uveitis:	[ ]
- Pediatrics/strabismus:	[ ]
- Neuro-ophthalmology:	[ ]
- Ocular pathology:	[ ]
- Ocular genetics:	[ ]
- Low vision rehabilitation:	[ ]
- Oculoplastic:	[ ]
- Undecided:	[ ]
Which province of Saudi Arabia are you planning for your future practice after completing your training:
**(Please choose one as first choice and one as a second choice)**	
	First choice	Second choice
Makkah		
Madinah		
Riyadh		
Eastern		
Assir		
Qassim		
Jizan		
Tabouk		
Najran		
Baha		
Hail		
North borders		
Jouf		
Unsure		
Are you interested in providing refractive surgery in the future: Choose One
- Yes	[ ]
- No	[ ]
- Unsure	[ ]
Are you planning for private practice:
- Yes	[ ]
- No	[ ]
If yes, specify:
- Full-time	[ ]
- Part-time	[ ]
How much time you will preserve for research:
- 0%	[ ]
- 25%	[ ]
- 50%	[ ]
- 75%	[ ]
- 100%	[ ]
- Unsure	[ ]

The data were categorized by gender, and the influence of gender on outcome was assessed in a univariate fashion using the Chi-square or Fisher exact test when appropriate. A *P*-value of 0.05 or less was considered statistically significant.

This study was a survey review and an institutional review board was obtained by the Department of Surgery.

## RESULTS

Of 85 residents surveyed, 68 (80%) responded to the survey. Fifty-six respondents (82.4%) were male and 12 (17.6%) were female. Thirty percent of the respondents were enrolled in Year 1, half of the residents were trained in Middle province. The demographic characteristics of the respondents are summarized in Table 2. The female respondents were significantly younger (*P* < 0.05) [Table 2].

**Table 2:** Demographic characteristics of respondents to the survey on ophthalmology career choice

Respondents	Total (%)	Male (%)	Female (%)	Chi-square statistic	*P*-value
	68 (100)	56 (82.4)	12 (17.6)		
Age					
<25	11 (16.1)	5 (8.9)	6 (50.0)	13.49	0.003^**^
25–29	36 (53.0)	33 (58.9)	3 (25.0)		
30–34	18 (26.5)	16 (28.6)	2 (16.6)		
35–39	3 (4.4)	2 (3.6)	1 (8.3)		
Province of training					
Western	16 (23.5)	10 (17.9)	6 (50)	7.37	0.118
Middle	29 (42.6)	27 (48.2)	2 (16)		
Eastern	20 (29.4)	16 (28.6)	4 (33)		
South	1 (1.5)	1 (1.8)	0		
Unsure	2 (2.9)	2 (3.6)	0		
Academic rank						
R1	30 (44.1)	22 (39.3)	8 (66.7)	3.16	0.368
R2	10 (14.7)	9 (16.1)	1 (8.3)	
R3	15 (22.1)	13 (23.2)	2 (16.7)	
R4	13 (19.1)	12 (21.4)	1 (8.3)	

R1 denotes a first year ophthalmology resident, R2 denotes a second year ophthalmology resident, R3 denotes a third year ophthalmology resident, R4 denotes a fourth year ophthalmology resident, *P*<0.05 was statistically significant

The majority of the residents (76.5%) indicated plans to pursue a part-time private practice [Table 3]. The remainder were either considering full-time private practice (8.8%) or undetermined (14.7%) [Table 3]. Most respondents (77%), regardless of gender, expressed an interest in refractive surgery [Table 3]. When questioned about their intentions of being involved in post-residency research, 75% answered that they plan to allocate at least 25% of their time.

**Table 3:** Survey responses to questions about future practice plans among Saudi ophthalmology residents

Question	Total no. (%)	Malesno. (%)	Femalesno. (%)	Chi-square statistic	*P*-value
	68 (100)	56 (82.4)	12 (17.6)		
Planning for private practice					
Unsure	10 (14.7)	8 (14.3)	2 (16.6)	0.046	0.977
Full time	6 (8.8)	5 (8.9)	1 (8.3)		
Part time	52 (76.5)	43 (76.8)	9 (75.0)		
Interested in refractive surgery	52 (76.5)	43 (76.8)	9 (75.0)	0.02	0.89
Time allocated to research					
0	3 (4.5)	3 (5.4)	0	1.61	0.204
25	37 (55.2)	32 (57.1)	5 (45.5)		
50	11 (16.4)	10 (17.9)	1 (9.1)		
75	3 (4.5)	1 (1.8)	2 (18.2)		
Unsure	13 (19.4)	10 (17.9)	3 (27.3)		

No. denotes number of respondents, *P*< 0.05 was statistically significant

All respondents were certain about their first preference regarding locale of practice. The majority (76%) preferred to practice in an urban setting in cities such as Riyadh, Makkah, Jeddah and the Eastern area. Only 18% were inclined to serve relatively less-developed regions such as Madinah, Asir, Jizan, Baha, Hail and Quassim [Table 4].

**Table 4:** Distribution for those who are planning to work at a private practice by location in Saudi Arabia

Place of practice	Total no. (%)	Maleno. (%)	Femaleno. (%)	Chi-square statistic	*P*-value
	58 (100)*	48 (82.8)	10 (17.2)		
Major	48 (82.8)	38	10		
Makkah and	15 (25.8)	13 (27.0)	2 (20.0)	3.37	0.185
Jeddah						
Riyadh	20 (34.5)	17 (35.4)	3 (30.0)			
Eastern Province	13 (24.4)	8 (16.6)	5 (50.0)			
Minor	10(17.2)	10	-		
Madinah	2 (3.4)	2 (4.2)	-		
Asir	3 (5.1)	3 (6.3)	-		
Jizan	2 (3.4)	2 (4.2)	-		
Baha	1 (1.7)	1 (2.1)	-		
Hail	1 (1.7)	1 (2.1)	-		
Qassim	1 (1.7)	1 (2.1)	-		

* Not all respondents preferred to work in a private setting as seen in Table 3 above

**Figure 1:**
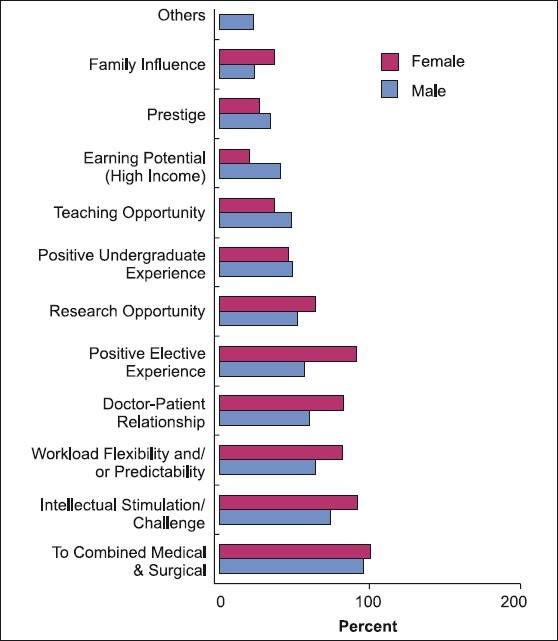
Responses to “What were the main factor influencing your Decision to pursue an ophtalmolagy residency training program”

The main factor influencing the decision to pursue ophthalmology training was the ability to combine medicine and surgery for 97% of the respondents. Intellectual stimulation (78%), workload flexibility and predictability (67%), ability to establish a good doctor-patient relationship (64%), positive elective experience (64%) and research opportunity (54%) were also commonly cited [Figure 1]. When comparing male residents with female residents, males were more inclined toward high earning potential (40.9% vs. 20%) and teaching opportunities (47.8% vs. 36.4%), while female residents were influenced more by positive elective experience (91% vs. 57%), doctor–patient relationship (82% vs. 60%) and workload flexibility and predictability (82% vs. 64%) [Figure 1].

**Figure 2:**
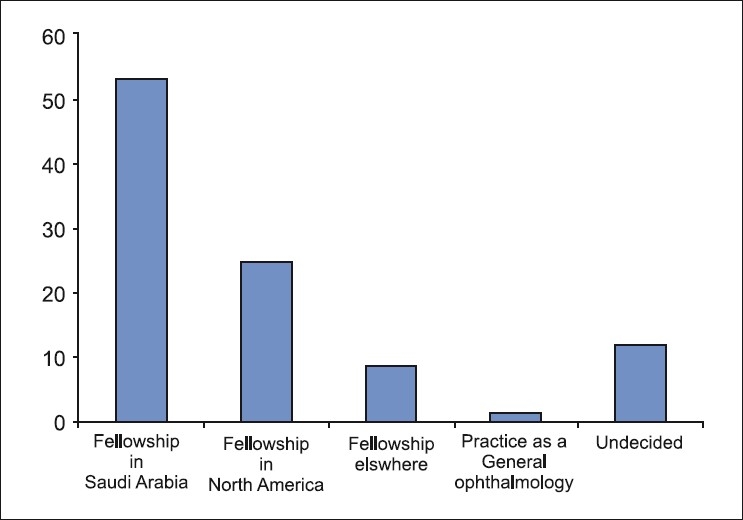
Responses to “What are your future plans after training”

**Figure 3:**
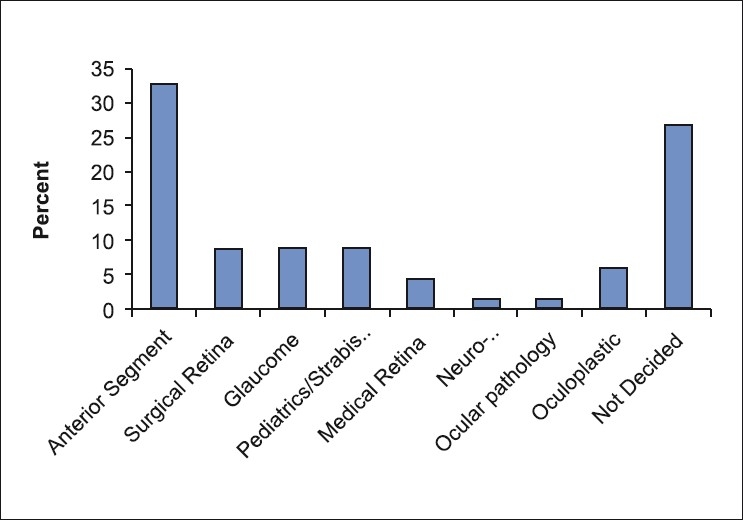
Responses ot “If you are considering fellowship which areas are you considering”

In response to future plans after completion of ophthalmology residency, more than half (53%) of the respondents indicated that they preferred to pursue a fellowship in Saudi Arabia (53%) and one-third of the respondents wished to be trained in North America (25%) or elsewhere overseas (9%) [Figure 2]. Only 15% wished to practice as a general ophthalmologist. When asked about their preferred subspecialty training [Figure 3], anterior segment was the most popular choice (33%), whereas surgical retina, glaucoma and pediatric ophthalmology were equally favored (9% each). Thirty percent of the residents were undecided at the time of the survey.

**Figure 4:**
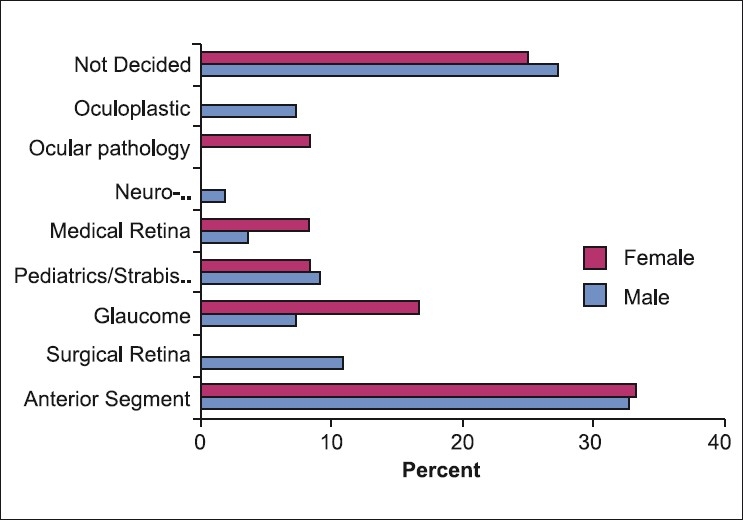
Responses to “If you are considering fellowship training, which areas are you considering by gender”

When comparing male and female residents in the preference of certain subspecialties [Figure 4], male residents were more likely to plan on pursuing surgical retina (11% vs. 0%) and oculoplastics (7% vs. 0%) while female residents were more interested in ocular pathology (8.3% vs. 0%) and glaucoma (17% vs. 7%). Anterior segment was the most favored subspecialty for both sexes and was distributed equally (34 vs. 33%). No statistically significant results were found between males and females in regard to province of training, plans for private practice, preferred place for future practice, interest in research and refractive surgery.

## DISCUSSION

The future trends in ophthalmic practice in Saudi Arabia will be shaped by multiple factors, including epidemiology of ophthalmic diseases, the current workforce, availability of resources and accessibility to new technologies. However, one of the most important factors include the career goals and the motivation of the current generation of residents in this field, which has been grossly overlooked in the literature.^5^ This current study provides new insight into the trends and factors influencing the future practice of junior ophthalmology residents in Saudi Arabia.

The KKESH has provided the majority of tertiary care in Saudi Arabia, treating more than 100,000 outpatients and 7,000 inpatients annually.^1^ Because of this overwhelming patient load, the government of Saudi Arabia has been trying to distribute ophthalmology services across the country by encouraging the development of private centers where ophthalmologists can work part-time at government facilities.^1^ In the current study, more than half of the ophthalmology residents surveyed answered that they plan to pursue a full-time private practice. Additionally, the majority of residents wished to stay in urban centers such as Jeddah, Riyadh and Eastern provinces, while only 18% were planning to serve rural areas. Only 15% of the current residents were planning on directly entering the workforce after training as a comprehensive ophthalmologist, whereas a significant number, both males and females, were interested in elective refractive surgery. This result implies that there is a trend toward greater subspecialization, which will be detrimental to the currently rising demand for primary eye care in rural areas. It may be necessary for the Saudi government to encourage the development of rural centers and attract comprehensive ophthalmologists to provide routine eye care services for the general population.

The most popular choice for fellowship training was anterior segment (33%), in contrast to other subspecialties including oculoplastics (6%), whose popularity has been rising in North America.^5^,^6^ This may be explained by the fact that the Saudi Arabian government has allocated a substantial portion of material and personnel resources to the anterior segment.^7^ This allocation was due to the 1984 survey that reported that more than 70% of the cases of blindness were due to cataract and corneal disease.^3^ Additionally, the once-overwhelming demand for oculoplastics has been reduced due to the continued aging and death of elderly patients affected by endemic trachoma.^3^ This reveals that a perceived favorable job market may be a significant motivational factor predicting the trend of fellowship training. A similar result has been shown in previous studies in the United States.^5^

Unfortunately, the disparity between the needs of the public and preferred subspecialty among residents was evident in our study. For example, glaucoma was not the most popular specialty, especially among male residents, despite the fact that nearly one out of four cases of blindness in Saudis older than 60 years is caused by glaucoma,^8^ with a 15% incidence in this age group. Pseudoexfoliation, a significant risk factor of open-angle glaucoma, is also common in Saudis, with an overall prevalence of 10% in the population.^8^ This raises concerns about the provision of adequate ophthalmic care for patients with advanced glaucoma.

As many as 75% of the current ophthalmology residents plan to allocate at least 25% of their time to research, among which 30% were planning to devote at least half of their time to research activities. In fact, ophthalmic research is actively being pursued in Saudi Arabia, providing giving new insight into the treatment of important ophthalmic disorders, in particular to genetic disorders that are common in Saudi Arabia due to the high prevalence of consanguinity.

Our study shows that the primary motivating factor that influenced the decision to pursue ophthalmology training was not only the inherent nature of ophthalmology but also the positive elective experience and perceived doctor–patient relationships, especially among female residents. This demonstrates the importance of clinical rotations and good mentorship in career decision making.^9^,^10^ Likewise, residency programs may be limiting exposure to comprehensive training in favor of certain subspecialty rotations, possibly due to the limited number of full-time comprehensive faculty in the university setting. This lack of faculty combined with greater perceived prestige and research opportunities associated with subspecialty likely further limits exposure to comprehensive ophthalmology.^5^ In addition, our study shows the rising popularity of refractive surgery among current residents, highlighting the influence of greater economic rewards in certain subspecialties.^10^

There are several limitations to our study. First, the limitation inherent in a cross-sectional survey makes it difficult to make casual inferences. Although we have achieved a non-biased response with an 80% response rate, as many as 30% of the respondents were undecided about their subspecialty. This can be attributed to the fact that the majority of respondents were still early in their training (first year ophthalmology residents) and had limited exposure to each subspecialty. Therefore, it may be concluded that, had another time-frame been chosen, a different result may have been obtained as residents may change their future plans with further training and experience. Some may also fail to match in a certain fellowship program regardless of their preferences. The dominant male response may reflect the fact that majority of eye service Saudi physicians are males (61.7%).^11^ Nevertheless, the present study may serve as a platform for future research and repeated cross-sectional studies may be useful to generate and assess the future trends in ophthalmology in Saudi Arabia.

In summary, the trends in this study showed that a large number of current ophthalmology residents prefer to obtain fellowship training in Saudi Arabia, with some subspecialties preferred over others. Many residents plan to work in providing private care in an urban setting. The future career decisions of residents may be largely influenced by good mentors and clinical exposure during medical school or residency training, and the number of fellowship positions may depend on factors other than the needs of the country. Therefore, the identification of trends and factors that influence career choice of ophthalmology residents may be helpful for the provision of ophthalmology residency programs in order to provide adequate eye care to all citizens of Saudi Arabia.
